# Norovirus Genetic Diversity in Children under Five Years Old with Acute Diarrhea in Mozambique (2014–2015)

**DOI:** 10.3390/v14092001

**Published:** 2022-09-09

**Authors:** Jorfélia J. Chilaúle, Benilde Munlela, Janet Mans, Victor V. Mabasa, Selma Marques, Adilson Fernando Loforte Bauhofer, Graziela Jane, Elda Anapakala, Fernanda Oliveira, Idalécia Cossa-Moiane, Esperança Guimarães, Júlia Sambo, Diocreciano Matias Bero, Assucênio Chissaque, Nilsa de Deus, Maureen B. Taylor

**Affiliations:** 1 Instituto Nacional de Saúde (INS), EN1, Bairro da Vila-Parcela n° 3943, Distrito de Marracuene, Maputo 264, Mozambique; 2 Department of Medical Virology, Faculty of Health Sciences, University of Pretoria, Pretoria 0001, South Africa; 3 Instituto de Higiene e Medicina Tropical (IHMT), Universidade Nova de Lisboa, Rua da Junqueira 100, 1349-008 Lisboa, Portugal; 4 Hospital Militar de Maputo (HMM), Rua Samuel D. Khumbula Maputo, Maputo 592, Mozambique; 5 Institute of Tropical Medicine (ITM), 2000 Antwerp, Belgium; 6 Departamento de Ciências Biológicas, Universidade Eduardo Mondlane, Av. Julius Nyerere–Campus Universitário, Maputo 257, Mozambique

**Keywords:** norovirus, genotypes, children, acute diarrhea, Mozambique

## Abstract

Norovirus (NoV) is the second most important cause of viral diarrheal disease in children worldwide after rotavirus and is estimated to be responsible for 17% of acute diarrhea in low-income countries. This study aimed to identify and report NoV genotypes in Mozambican children under the age of five years with acute diarrhea. Between May 2014 and December 2015, stool specimens were collected within the Mozambique Diarrhea National Surveillance (ViNaDia) and tested for NoV genogroups I (GI) and II (GII) using conventional reverse transcriptase-polymerase chain reaction (RT-PCR). Partial capsid and RNA-dependent RNA polymerase (RdRp) nucleotide sequences were aligned using the Muscle tool, and phylogenetic analyses were performed using MEGA X. A total of 204 stool specimens were tested for NoV. The detection rate of NoV was 14.2% (29/204). The presence of NoV was confirmed, by real-time RT-PCR (RT-qPCR), in 24/29 (82.8%) specimens, and NoV GII predominated (70.8%; 17/24). NoV GII.4 Sydney 2012[P31] was the predominant genotype/P-type combination detected (30.4%; 7/23). This is the first study which highlights the high genetic diversity of NoV in Mozambican children and the need to establish a continuous NoV surveillance system.

## 1. Introduction

Globally, norovirus (NoV) is the second most important cause of viral diarrheal disease in children under five years old, after rotavirus infection [1]. A systematic review showed that NoVs are responsible for 12% of severe diarrhea and up to 200,000 deaths in children under five years old in low-income countries [2]. However, more recent meta-analysis studies estimate that NoV is responsible for 16–18% of acute diarrhea globally in children under five years old [3,4].

NoVs are non-enveloped viruses classified in the *Caliciviridae* family, genus *Norovirus*, with a positive sense single-stranded RNA (ssRNA) genome. NoVs are classified into ten genogroups (GI to GX) and 49 genotypes, based on amino acid sequence diversity in the complete VP1 capsid protein [5]. The NoV genogroups that infect humans are GI, GII, GIV, GVIII and GIX [5]; however, GII and GI are the predominant causes of infection worldwide [6,7,8,9].

Data from 19 studies in 14 African countries on children under five years of age, hospitalized with acute gastroenteritis, showed an overall norovirus prevalence of 13.5% (961/7141; 95% CI 12.7–14.3), with a range of 0.8–25.5% [10].

In Mozambique, two studies carried out in children under five years in the Manhiça district showed the circulation of NoV in the southern region of the country [11,12]. However, the genotypes were not identified, thus there is a lack of NoV genotyping information in Mozambique, and no routine surveillance system has been established. Therefore, in 2014, the *Instituto Nacional de Saúde* (INS)—Mozambique implemented the ViNaDia with the aim of estimating the burden of rotavirus and other enteric agents in children with diarrheal diseases in three regions (south, center and north) of Mozambique. In addition, this surveillance system allows the determination of the prevalence and molecular epidemiology of NoV. The aim of this study is to describe the epidemiology and circulating genotypes of NoV in children under five years old with diarrhea in these three regions of Mozambique.

## 2. Materials and Methods

### 2.1. Study Population and Stool Sample Collection

Between May 2014 and December 2015, a cross-sectional study in children under five years old with acute diarrhea was conducted. Acute diarrheal disease was defined as the passage of three or more loose or liquid stools per day in less than 7 days [13]. A single stool sample was collected from each participant enrolled at one of the six ViNaDia sentinel sites [13,14].

### 2.2. Laboratory Testing

#### 2.2.1. Detection and Molecular Characterization

Viral RNA was extracted from 10% fecal suspensions (RNase/DNase free water) using a QIAamp Viral RNA Mini Kit (QIAGEN, Valencia, CA, USA), according to the manufacturer’s recommendations. Complementary DNA (cDNA) synthesis was performed using the High-Capacity cDNA Reverse Transcription kit (Applied Biosystems^TM^, Foster City, CA, USA) according to the manufacturer’s instructions.

NoV was initially detected by RT-PCR, using sets of genogroup-specific primers for GII (Mon 431/433) and GI (Mon 432/434) that target the RdRp (region B). Previously described PCR conditions were used [15], and the PCR products were analyzed on a 1.5% agarose gel stained with ethidium bromide and observed under ultraviolet light.

Aliquots of positive specimens were sent to South Africa (Department of Medical Virology, University of Pretoria) for confirmation using RT-qPCR, which targets the ORF1/2 junction. RNA was extracted from 10% fecal suspensions using the semi-automated NucliSENS EasyMAG Instrument (bioMérieux, Marcy l’Etoile, France). RT-qPCR was performed to detect GI and GII using the QuantiFast Pathogen RT-PCR + IC kit and published primers and probes (Table 1) [16,17]. The NoV strains detected by RT-qPCR were genotyped using primers specific for the partial RdRp (region A) [18] and partial capsid (region C) [19] (Table 1), as previously described [20], except that Emerald-Amp MAX PCR master mix (Takara Bio Inc., Shiga, Japan) was used. PCR-positive products were purified (DNA Clean & Concentrator-25 Kit, Zymo Research, Irvine, CA, USA) and sequenced using the Big Dye Terminator v3.1 kit (Applied Biosystems, Waltham, MA, USA).

#### 2.2.2. Phylogenetic Analysis

BioEdit Sequence Alignment Editor was used for sequence alignment. Phylogenetic analysis was performed using MEGA X [21]. The genetic distance was calculated by the Tamura–Nei + gamma distributed (T93+G) for GI capsid gene, Tamura 3-parameter + gamma distributed + invariant sites (T92+G+I) for RDRP gene, Kimura-2-parameter + gamma distributed (K2+G) for GII capsid and RDRP model using the maximum-likelihood method, with 1000 bootstrap replicates to estimate branch support. The nucleotide partial sequences determined in this study were submitted to GenBank with the following accession numbers: GI RdRp (ON951678–ON951684), GI capsid (ON951685–ON951691), GII RdRp (ON951692–ON951705) and GII capsid (ON951706–ON951721).

### 2.3. Data Management and Analyses

Socio-demographic and clinical information of the children were double entered into an Epi Info^TM^ 3.5.1 (CDC, Atlanta, GA, USA, 2008) database, after which a single cleaned dataset was used. Descriptive statistics were used to summarize the data in frequencies; Chi-square and Fisher’s exact test were used to identify socio-demographic variables associated with NoV detection. Data were analyzed in IBM SPSS software (Statistical Package for the Social Science, Armonk, NY, USA: IBM Corp, 2011, version 26.0, Chicago, IL, USA).

### 2.4. Ethical Approval

The National Bioethical Committee for Health of Mozambique approved the study protocol (IRB00002657, reference N^o^: 348/CNBS/13). Parents or guardians of eligible children provided informed consent after receiving information about the purpose of the study.

## 3. Results

### 3.1. Norovirus Prevalence

During the study period, 204 stool specimens from children under five years old were tested for NoV. The majority of children (87.3%; 178/204) were under two years old and 57.8% (118/204) were male (Table 2). Most of the tested samples were from Maputo City (56.7%; 116/204), followed by Nampula (36.8%; 75/204). NoV was detected in 14.2% (29/204) of children by RT-PCR; infections were observed in all age groups and both genders, with comparable detection in age groups 0 to 11 months (16/100; 16.0%) and 12 to 23 months (12/78; 15.4%) and higher detection in males (22/118; 18.6%; *p* = 0.034) (Table 2).

Regarding clinical features, the NoV infection rate was similar between children with and without vomiting (14.3%, 19/133 versus 14.5%, 10/69, respectively), similar between human immunodeficiency virus (HIV)-positive and HIV-uninfected children (17.7%, 3/17 versus 15.5%, 21/134, respectively) and lower in children with fever (10.0%, 6/60 versus 15.4%, 21/136), although this was not statistically significant (Table 2). The proportion of NoV infection varied among the three groups of water sources (tap in the house, public tap and well) but differences were not statistically significant (Table 2).

### 3.2. Norovirus Genotyping

**Figure 1:**
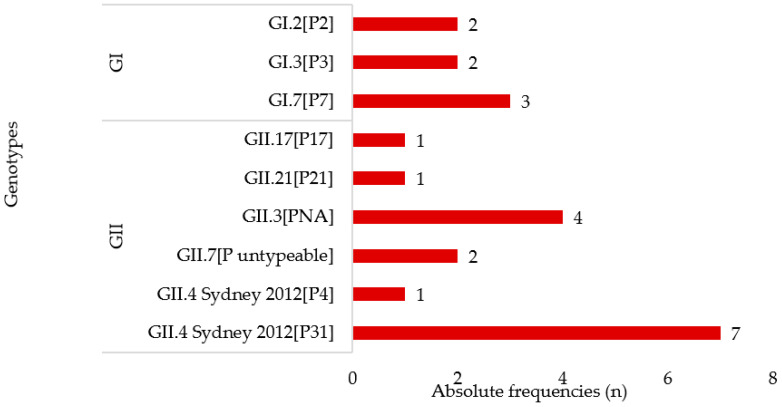
Distribution of norovirus genogroups and genotypes in samples collected from 23 children.

RT-qPCR confirmed norovirus presence in 82.8% (24/29) of samples that previously tested positive by conventional RT-PCR. The discrepancy in NoV detection rates between the conventional RT-PCR and RT-qPCR could possibly be ascribed to sample degradation during storage and subsequent transport to South Africa. In addition, nucleotide sequence differences could allow detection with one assay (B region) but not with another (ORF1/2 junction). NoV GII was the predominant genogroup (70.8%; 17/24). Overall, 95.8% (23/24) of the strains were genotyped by nucleotide sequence analysis. The partial RdRp and capsid sequence analysis revealed the predominance of GII.4 Sydney 2012[P31] (30.4%; 7/23), followed by GII.3[PNA] (17.4%; 4/23) and GI.7[P7] (13.0%; 3/23) (Figure 1).

### 3.3. Phylogenetic Analyses

#### 3.3.1. VP1 Gene

**Figure 2:**
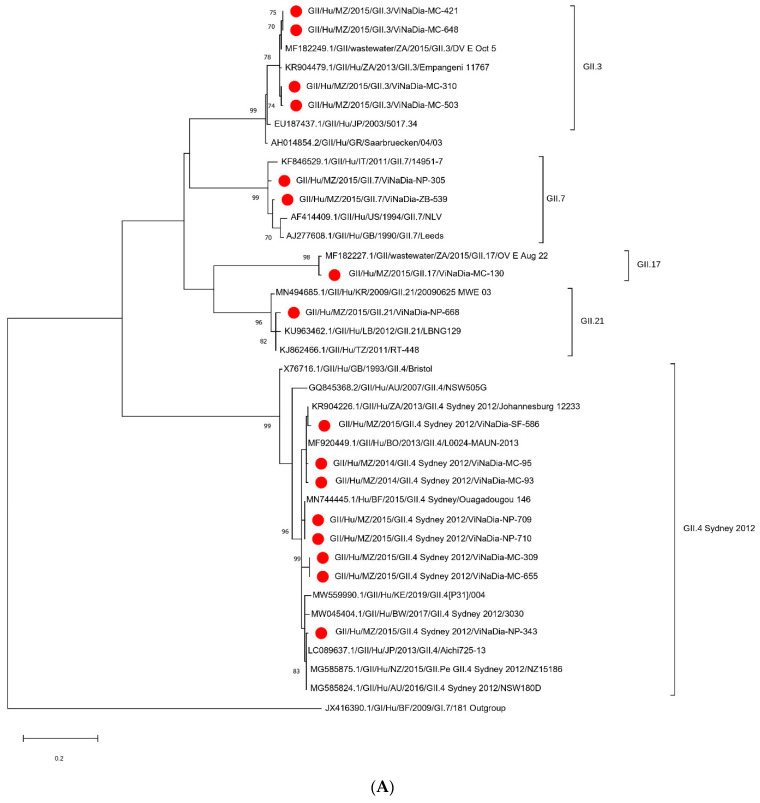

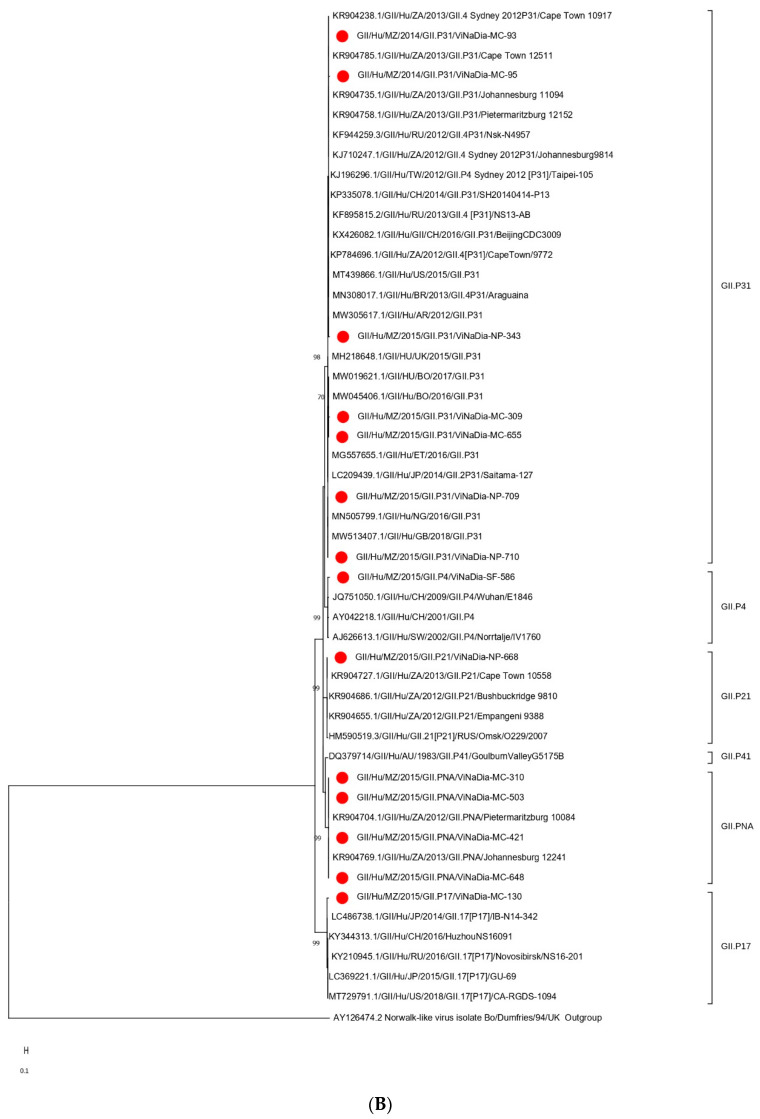
(**A**) Maximum-likelihood phylogenetic tree of partial VP1 gene (region C), representing 16 NoV GII strains comprising five genotypes from Mozambique, and (**B**) maximum-likelihood phylogenetic tree of partial RdRp gene (region A), representing 14 NoV strains depicting five P-types from Mozambique. Bootstrap support of >70% is indicated.

**Figure 3:**
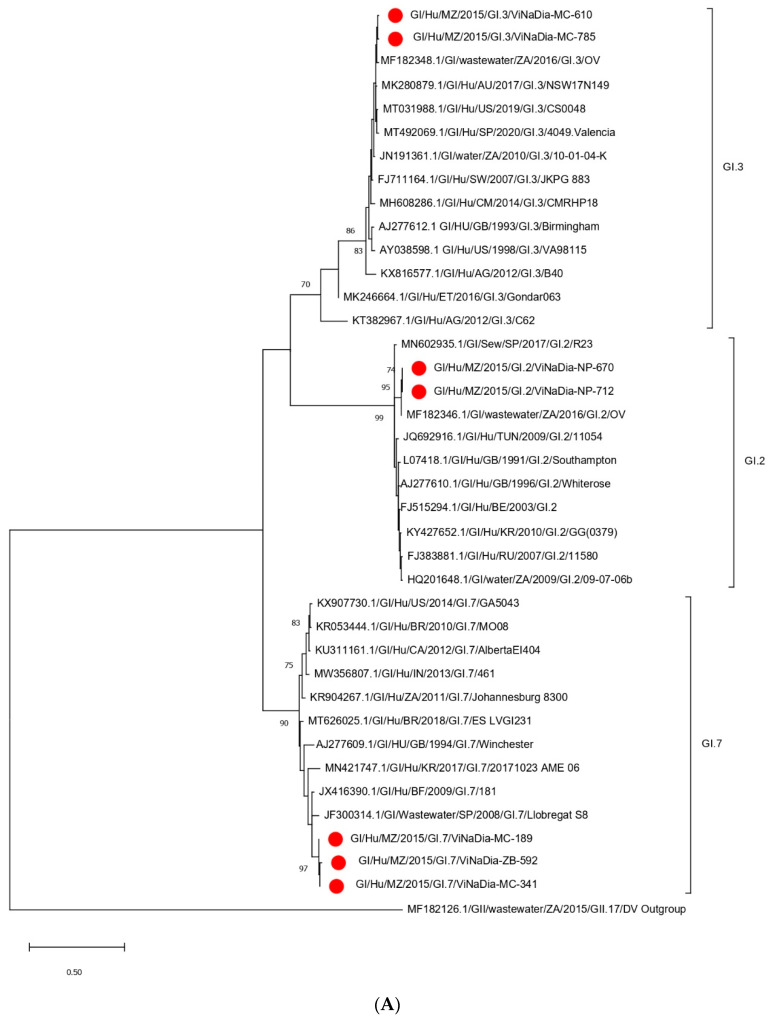

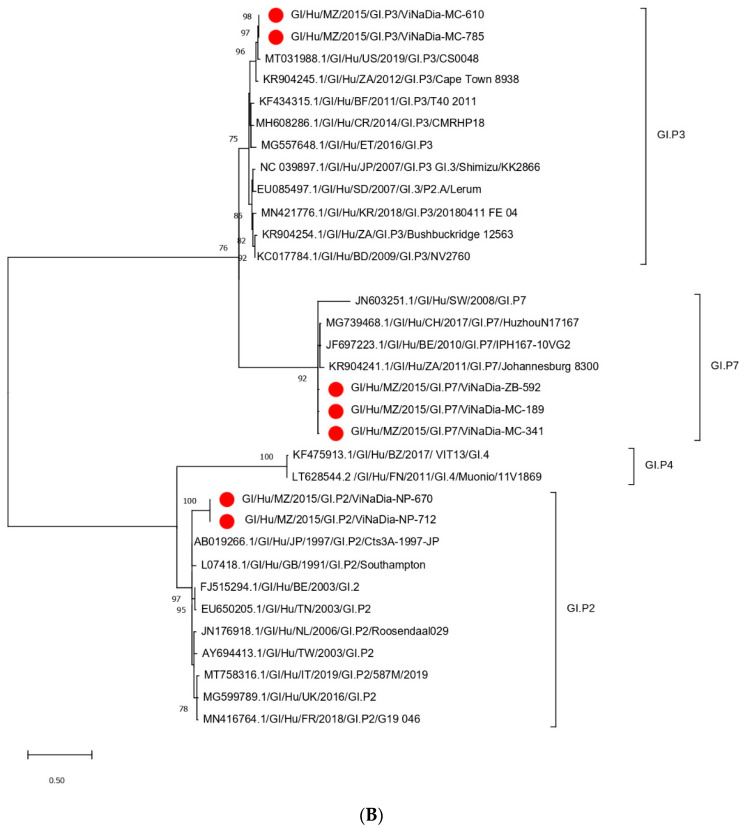
(**A**) Maximum-likelihood phylogenetic tree of partial VP1 gene (region C), representing seven NoV GI strains and three genotypes from Mozambique, and (**B**) maximum-likelihood phylogenetic tree of partial RdRp gene (region A), representing seven NoV GI strains and four P-types from Mozambique. Bootstrap support of >70% is indicated.

Phylogenetic analysis of the partial VP1 gene sequence of the 23 identified strains showed that 16 sequences belonged to GII (Figure 2A) and 7 belonged to GI (Figure 3A). Within GII, genotypes GII.4 Sydney 2012 (eight strains), GII.3 (four strains), GII.7 (two strains), GII.17 (one strain) and GII.21 (one strain) were identified. Three GI genotypes were characterized; namely, GI.7 (three strains), GI.3 (two strains) and GI.2 (two strains). The majority of GII.4 Sydney 2012 Mozambican strains clustered with African strains (Botswana, South Africa and Burkina Faso) and Asian strains (Japan) (Figure 2A). The GII.3 Mozambican NoV strains clustered close to strains detected in humans and wastewater in South Africa. One GII.7 strain clustered with a strain from Italy and the other GII.7 formed a subcluster with strains from the United States of America (USA) and Gabon. The GI.3 and GI.2 Mozambican strains were closely related to wastewater strains detected in South Africa. The GI.7 Mozambican strains formed a distinct subcluster between them, and they were closely related to a wastewater strain detected in Spain (Figure 3A).

#### 3.3.2. RdRp Gene

Phylogenetic analysis of the partial RdRp gene sequence of the 21 identified strains showed that 14 sequences belonged to GII (Figure 2B) and 7 belonged to GI (Figure 3B). Within GII, P-types GII.P31 (seven strains), GII.PNA (four strains), GII.P4 (one strain), GII.21 (one strain) and GII.17 (one strain) were identified, and, within GI, P-types GI.P7 (three strains), GI.P3 (two strains) and GI.P2 (two strains) were detected. The GII.P31 formed four distinct subclusters with strains from Africa (South Africa, Botswana, Gabon) and Argentina. The GII.P4 strain from the central Mozambique region was closely related to a Chinese strain (JQ7510560). All GII.PNA strains were closely related to South African strains (Figure 2B). The GI.P7, GI.P3 and GI.P2 Mozambican strains formed three subclusters between them and were related to strains detected in South Africa, USA and Japan, respectively (Figure 3B).

## 4. Discussion

This study aimed to determine the epidemiology and genotypes of NoV in children under five years old with diarrhea in all three regions of Mozambique. Approximately 3 in 20 children were infected (14.2%). The proportion observed is higher than a previous study in diarrhea cases conducted in a rural setting in the Manhiça district, southern Mozambique (4.2%; 33/784; *p*-value < 0.001) [12]. This difference can be explained by the sentinel site location (urban versus rural site) and the sampling period. This difference suggests an increased occurrence of NoV in children with diarrhea over the years; the same has been observed in Malawian children [22,23], of whom a previous study identified NoV in 6.5% and later in 11.3%, both before the rotavirus vaccine introduction.

NoV infections were detected in all age groups, but a higher rate of infection was observed in children younger than 12 months. These findings are consistent with previous findings that showed that NoV infections usually occur in early childhood [24]. NoV GI and GII were the identified genogroups, with GII being the most common (70.8%). A similar result was observed in previous studies in Malawi [25], Zambia [26], Ghana [6], Brazil [8,9] and other countries in Africa where GII strains detection rates range from 71% to 100% with an average of 84.1% [10].

The partial sequence results of the 23 study strains showed the circulation of nine NoV genotypes during the study period, suggesting a significant genetic diversity of NoV in Mozambique. Overall, the capsid genotype GII.4 Sydney 2012 was the most common in Mozambique, reflecting global trends [10,27]. NoV GII.4 was reported to be the most prevalent genotype in Africa at 54.1%, followed by GII.3 at 12.2% [10], consistent with our findings. Norovirus GI was observed in one-third of the samples. GI strains are common causes of foodborne and waterborne outbreaks [28].

Regarding the partial RdRp gene, few studies reported RdRp typing data. In this study, the GII.P31 (previously named GII.Pe) was the most identified P-type, which is consistent with previous studies from the USA [10,20,27,29,30].

The GII.4 Sydney 2012 strains formed several subclasses, some of them clustered within the same province, suggesting that strains that circulated in the country in the same year were relatively different. The Maputo City and Nampula provinces showed more diversity in the genotype distribution and close relatedness with strains from non-neighboring countries such as Japan, Lebanon, Burkina Faso, USA and China. These Mozambican provinces have the highest economic and political activity with the most population flow and major development in the country [31]. Additionally, Nampula has the Nacala development corridor which stimulates cross-border trade, resulting in an increased movement of people in the geographic region [31]. However, other strains clustered close to viruses detected in neighboring southern African countries. The main limitations of this study were: (1) the limited number of samples tested; (2) the surveillance was hospital-based and focused on severe gastroenteritis; therefore, data on mild and asymptomatic infections are lacking; (3) the samples were from 2014–2015, so the current genotype circulation may be different; and (4) seasonality could not be evaluated due to the duration of study being less than 24 months.

## 5. Conclusions

This study showed the importance and genetic diversity of NoV mainly in Mozambican children under two years old. NoV occurs in children with acute diarrhea in all three regions of Mozambique. The identified genogroups were GI and GII. Genotype clustering suggests different sources of infection in each province. The study provides valuable baseline data of NoV occurrence and genetic diversity in Mozambique. The data emphasizes the need to educate caregivers about child health, hygiene and water treatment, especially for children under two years of age. The improved management of childhood diarrhea in health facilities is essential to reduce morbidity and mortality. Continued surveillance is required to identify infection trends and monitor virus evolution in the Mozambican community.

## Figures and Tables

**Table 1:** Primer sequences used for real-time RT-PCR, RT-PCR and genotyping of norovirus GI and GII.

Target	Norovirus	Primer	Sequence (5′–3′) *	Polarity	Location
Region A	Noroviruses	JV12Y [18]	ATA CCA CTA TGA TGC AGA YTA	+	4279–4299 ^‡^
		JV13I [18]	TCA TCA TCA CCA TAG AAI GAG	−	4585–4605 ^‡^
Region B	GI	Mon 432 [15]	TGG ACI CGY GGI CCY AAY CA	+	
		Mon 434 [15]	GAA SCG CAT CCA RCG GAA CAT	−	
	GII	Mon 431 [15]	TGG ACI AGR GGI CCY AAY CA	+	
		Mon 433 [15]	GAA YCT CAT CCA YCT GAA CAT	−	
Region C	GI	QNIF4 [16]	CGC TGG ATG CGN TTC CAT	+	5291–5308 ^†^
		G1SKF [19]	CTG CCC GAA TTY GTA AAT GA	+	5342–5361 ^†^
		G1SKR [19]	CCA ACC CAR CCA TTR TAC A	−	5653–5671 ^†^
	GII	QNIF2 [17]	ATG TTC AGR TGG ATG AGR TTC TCW GA	+	5012–5037 ^‡^
		G2SKF [19]	CNT GGG AGG GCG ATC GCA A	+	5046–5064 ^‡^
		G2SKR [19]	CCR CCN GCA TRH CCR TTR TAC AT	−	5367–5389 ^‡^

* Mixed bases in degenerate primers are as follows: Y = C or T; R = A or G; W = A or T; I = Inosine; N = any; S = C or G. ^†^ Location based on M87661. ^‡^ Location based on X86557.

**Table 2:** Socio-demographic and clinical characteristics of children under five years old with and without norovirus in Mozambique 2014–2015 (*n* = 204).

Characteristic	Number in Category/ Total Number of Samples (%)	Number of NoV-Positive Samples (%)	*p*-Value
Total Enrolled	204	29 (14.2)	
Sex			**0.034 ^a^**
Male	118/204 (57.8)	22/118 (18.6)	
Female	86/204 (42.2)	7/86 (8.1)	
Age group (months)			0.292 ^b^
0–11	100/204 (49.1)	16/100 (16.0)	
12–23	78/204 (38.2)	12/78 (15.4)	
24–59	26/204 (12.7)	1/26 (3.8)	
Province			0.400 ^b^
Maputo city	116/204 (56.7)	18/116 (15.5)	
Sofala	5/204 (2.5)	1/5 (20.0)	
Zambézia	8/204 (3.9)	2/8 (25.0)	
Nampula	75/204 (36.8)	8/75 (10.7)	
Vomiting			0.986 ^a^
Yes	133/202 (65.8)	19/133 (14.3)	
No	69/202 (34.2)	10/69 (14.5)	
Unknown/missing	2	0	
Fever			0.308 ^a^
Yes	60/196 (30.6)	6/60 (10.0)	
No	136/196 (69.4)	21/136 (15.4)	
Unknown/missing	8	2	
HIV Status			0.735 ^b^
Positive	17/149 (11.4)	3/17 (17.6)	
Negative	134/149 (88.6)	21/134 (15.7)	
Unknown/missing	53	5	
Source of drinking water			0.496 ^a^
Tap	113/197 (57.4)	14/113 (12.4)	
Public tap	40/197 (20.3)	8/40 (20.0)	
Well	43/197 (21.8)	6/43 (14.0)	
Purchased/bottled water	1/197 (0.5)	0	
Unknown/missing	7	1	
Diarrhea episodes			0.916 ^b^
3	81/204 (39.7)	12/81 (14.8)	
4–6	101/204 (49.5)	15/101 (14.8)	
≥7	22/204 (10.8)	2/22 (9.1)	

^a^ Chi-square test; ^b^ Fisher’s exact test. Bold: significant *p*-values.

## Data Availability

The data are available upon reasonable request from the corresponding author.
